# Development of tight junction-strengthening compounds using a high-throughput screening system to evaluate cell surface-localized claudin-1 in keratinocytes

**DOI:** 10.1038/s41598-024-53649-1

**Published:** 2024-02-09

**Authors:** Hiroki Sakamoto, Momoyo Nishikawa, Seigo Yamada

**Affiliations:** Research and Development Headquarters, Well-Being Research Laboratories, Lion Corporation, 100 Tajima, Odawara, Kanagawa 256-0811 Japan

**Keywords:** Membrane proteins, Tight junctions, Target identification

## Abstract

Tight junctions (TJs) are important factors constituting the physical barriers of the skin, and their suppression has been described in various conditions, such as aged skin and atopic dermatitis lesions. However, the methods for improving skin TJ function remain insufficient. Therefore, to obtain compounds that can improve TJ function, we developed a novel high-throughput screening system termed live-cell immunostaining to evaluate cell surface-localized claudin-1 (CLDN1) with high selectivity using normal human epidermal keratinocytes (NHEKs). Heparinoid and phospho-pyridoxal (p-Pyr), a metabolite of pyridoxine, were identified as hit compounds. In addition, heparinoid was strongly suggested to increase CLDN1 expression by inhibiting epidermal growth factor receptor signaling. By contrast, p-Pyr did not enhance CLDN1 expression, but it accelerated the translocation of CLDN1 to the cell surface. Finally, we confirmed that heparinoid and p-Pyr improved barrier function in NHEKs in a transepithelial electrical resistance assay. In conclusion, heparinoid and p-Pyr could potentially ameliorate skin conditions by improving TJ function.

## Introduction

Skin separates the body from the external environment, and the barrier function of the skin plays a crucial role in protecting against external stimuli and preventing water evaporation from the body^1^. The physical barriers of skin are the stratum corneum and tight junctions (TJs)^2^. The stratum corneum is the outermost layer of the epidermis, and it is mainly formed by corneocytes and intercellular lipids^3^. TJs are intercellular junctions in the second layer of the stratum granulosum that restrict the passage of substances such as water and larger molecules^4^. Furthermore, TJs are reported to inhibit the extension of nerve cells to the surface of the epidermis^5^, and it is suggested that healthy TJs help ameliorate pruritus. TJs contain various proteins, including transmembrane proteins (claudins [CLDNs], occludin [OCLN], and junctional adhesion molecules), and zonula occludens (ZOs), which are scaffold proteins that anchor transmembrane proteins^4^. In particular, CLDN1 is one of the main factors of TJs, and marked increases in transepidermal water loss and mortality associated with water loss have been observed in *CLDN1*-deficient mice^6^. Additionally, skin diseases associated with TJ dysfunction have been described. A marked reduction of CLDN1 in the non-lesional epithelium in patients with atopic dermatitis has been established^7^ and other research reports indicate that CLDN1 and ZO-1 expression in patients with atopic dermatitis is lower in the lesion area than in the non-lesion area^8^. In addition, *CLDN1* gene mutations are involved in neonatal sclerosing cholangitis (NISCH syndrome)^9^. Even in the absence of particular skin diseases, CLDN1 and OCLN expression decreases with age^10^, suggesting that TJs are involved in the age-related failure of skin.

In the present study, we aimed to discover compounds that can reinforce TJ function to improve skin health. TJ proteins such as CLDN1, OCLN, and CLDN4 exert their barrier effects upon localizing to the cell surface and interacting with neighboring cells. It is reported that CLDN1 localization is altered by various post-translational modifications including phosphorylation and nitration^11–13^. Therefore, although it is important to quantify the protein expression of CLDN1, selectively evaluating CLDN1 localization on the cell surface is more efficient for improving barrier function. Meanwhile, methods for quantifying total protein expression such as enzyme-linked immunosorbent assay (ELISA) are useful for high-throughput screening. Conversely, immunostaining, a representative method for evaluating cell surface-localized proteins, has limitations regarding quantitation and throughput.

## Results

### Screening system for the quantification of surface-localized CLDN1

**Figure 1:**
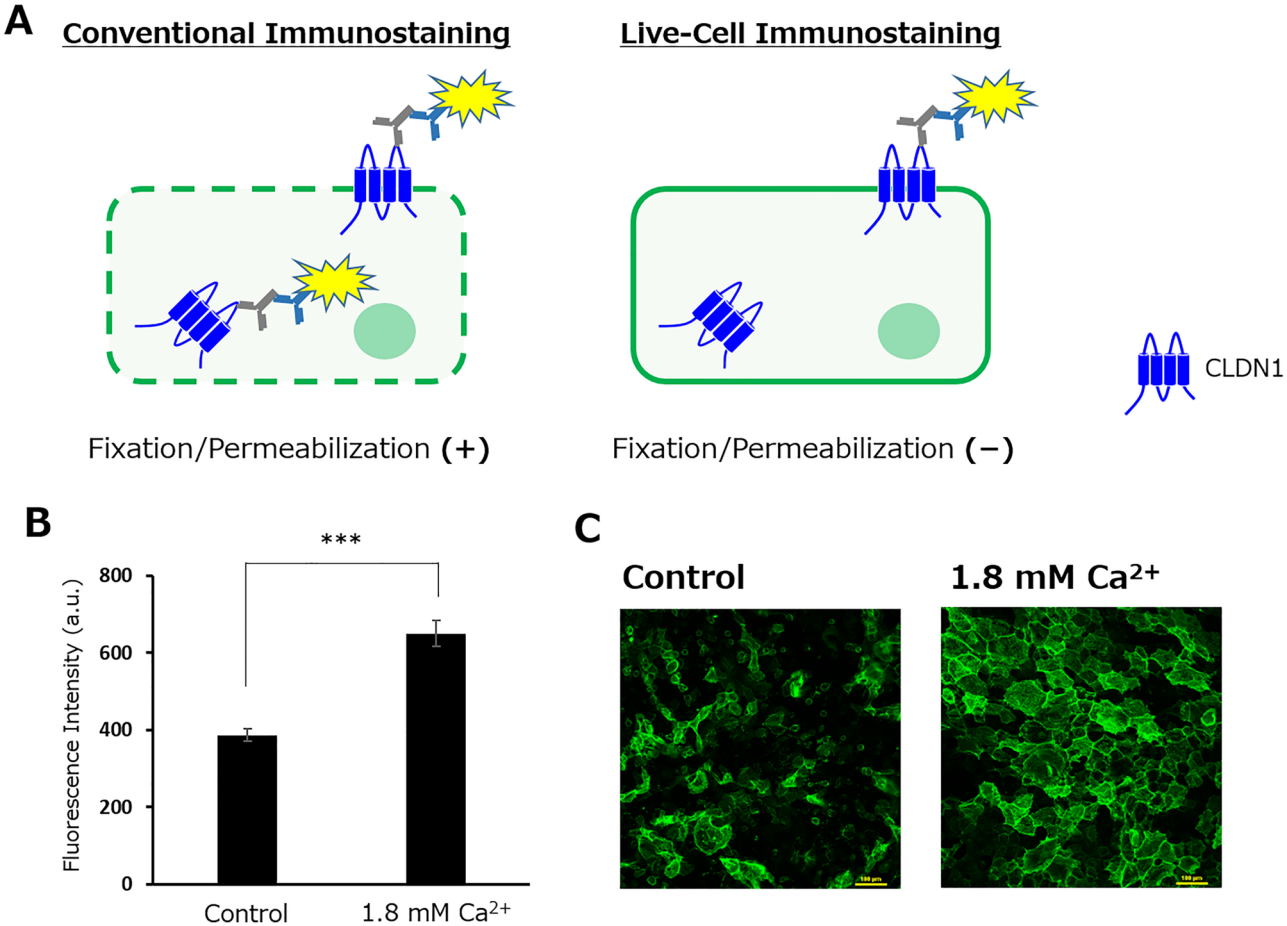
Live-cell immunostaining method to selectively detect surface-localized CLDN1. (**A**) Schematic diagram of each immunostaining method. (**B**) Fluorescence intensity and (**C**) microscopic images of NHEKs after CaCl_2_ treatment (24 h). Student’s *t*-test, ***p < 0.001 (n = 6). Scale bar: 100 μm.

To selectively detect CLDN1 localized on the cell surface, we used live-cell immunostaining, which does not include fixation and permeabilization steps, unlike conventional immunostaining systems (Fig. 1A), as a novel high-throughput system. In this method, plasma membranes are kept intact, and it is expected that anti-CLDN1 antibodies can only bind to cell surface-localized CLDN1. Therefore, we can measure surface-localized CLDN1 expression easily and efficiently using a microplate reader. The anti-CLDN1 antibody clone 7A5, which recognizes the second extracellular loop domain of CLDN1^14^, was used for this screening system. Because the fixation step was skipped, there were concerns about morphological changes and viability in cells; however, no changes were observed in normal human epidermal keratinocytes (NHEKs) because of their strong ability to adhere to culture vessels (Supplementary Fig. 1). Consequently, a significant enhancement in fluorescence intensity was observed 24 h after the addition of CaCl_2_ (Ca^2+^), which increases surface-localized CLDN1 levels^15^ (Fig. 1B). By performing the control assay in the presence and absence of each antibody, it was confirmed that the increase in fluorescence intensity does not depend on the nonspecific binding of fluorescent secondary antibody to Fc receptors on the plasma membrane (Supplementary Fig. 2). Additionally, the localization of CLDN1 to the plasma membrane of NHEKs was confirmed by microscopy (Fig. 1C). A slight fluorescence signal was observed from the intracellular fraction; however, treatment with cytochalasin D, which inhibits actin polymerization and macropinocytosis, enhanced the ratio of the fluorescence signal between the cell surface and cytoplasm (Supplementary Fig. 3). Thus, the intracellular signal was assumed to derive from CLDN1 originally localized on the cell surface. Therefore, this screening system was considered effective for assessing CLDN1 function.

### Compound screening using the live-cell immunostaining method

**Figure 2:**
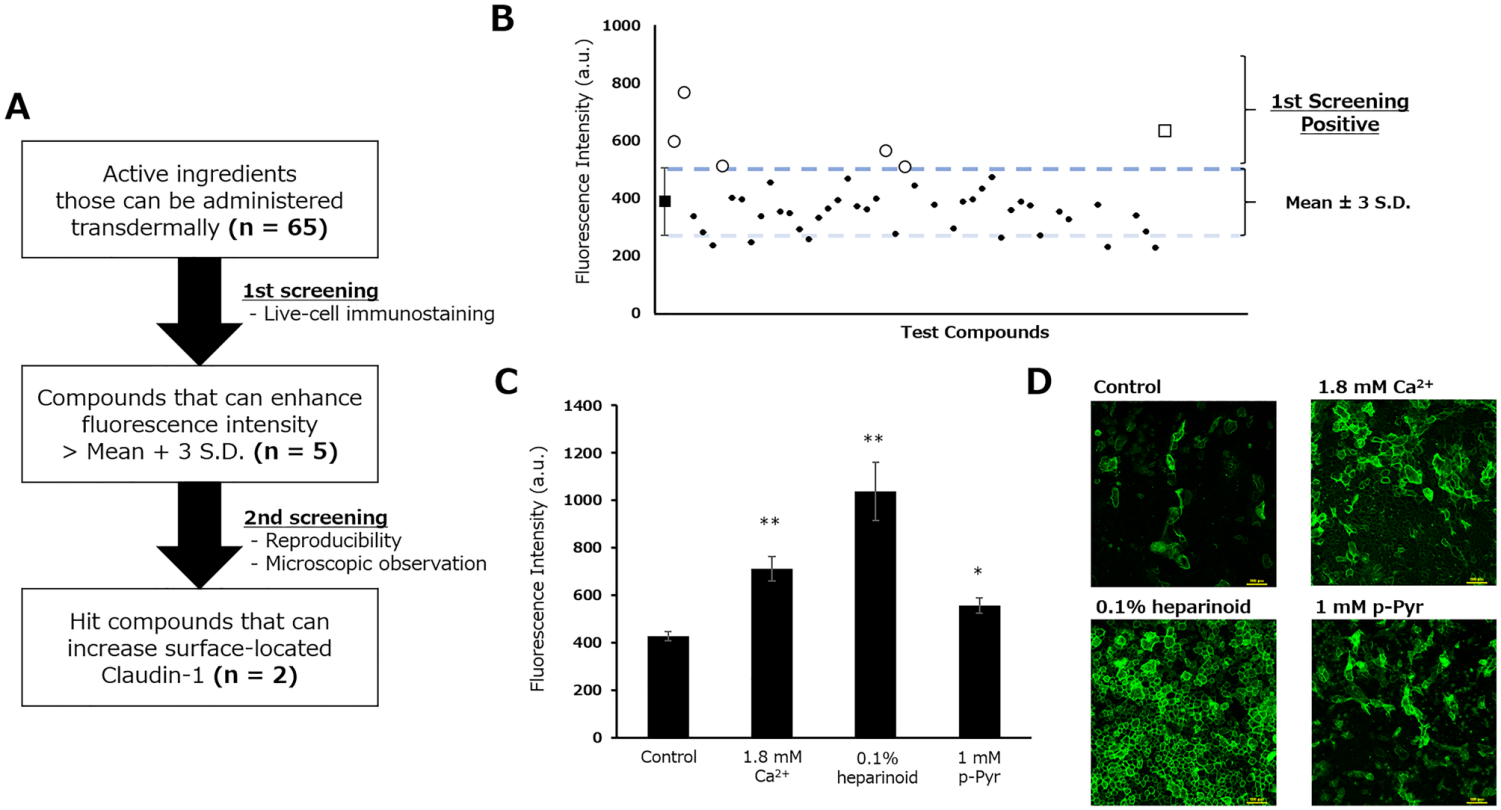
Compound screening using live-cell immunostaining. (**A**) Flowchart of screening. (**B**) First screening. Open circles and squares denote positive compounds and CaCl_2_, respectively. Some compounds with aberrant values were excluded. (**C**) Second screening. Compounds that reproducibly increased fluorescence intensity were acquired as hits. Dunnett’s test vs. Control, *p < 0.05, **p < 0.01 (n = 3). (**D**) Representative confocal microscopic images of CLDN1. Scale bar: 100 μm.

Using the developed screening system, we evaluated 65 samples mainly consisting of active ingredients that can be administrated transdermally (Fig. 2A, Supplementary table). Measurement was performed after 24 h of treatment with the compounds at concentrations of 1 mM or 0.1% w/v. A fluorescence intensity higher than the mean + 3 standard deviations of the non-treated control (n = 6) was set as the threshold for the first screening, and consequently, the list of compounds was narrowed to five candidates (Fig. 2B). In the second screening, compounds with poor reproducibility or the ability to increase surface-localized CLDN1 expression on microscopic observation were excluded, and finally, we acquired two hit compounds, namely heparinoid and phospho-pyridoxal (p-Pyr; Fig. 2C,D). In addition, heparinoid and p-Pyr did not exhibit cytotoxicity in a lactose dehydrogenase (LDH) assay or by morphological observation (Supplementary Figs. 4, 5). In particular, the heparinoid-induced increase in fluorescence intensity was remarkable, and the adhesion of cells to each other via CLDN1 was clearly observed.

### Action mechanisms of the hit compounds

**Figure 3:**
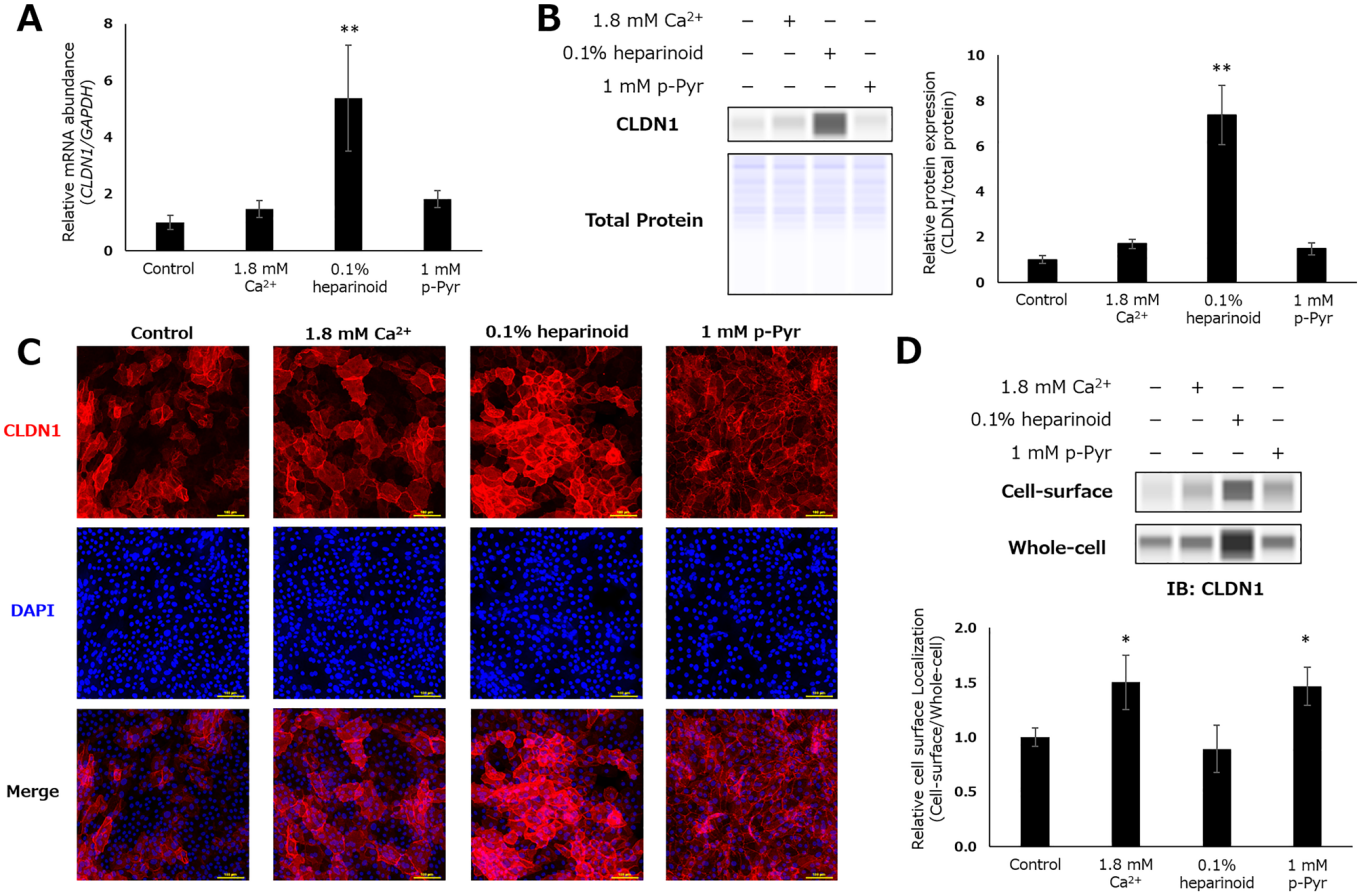
Alteration of CLDN1 expression following hit compound exposure in NHEKs. (**A**) Relative mRNA expression after 6 h of treatment. Dunnett’s test vs. Control, *p < 0.05, **p < 0.01 (n = 3). (**B**) Relative protein expression after 24 h. Dunnett’s test vs. Control, *p < 0.05, **p < 0.01 (n = 4). Original blots are presented in Supplementary Fig. 6. (**C**) Representative confocal microscopic images using conventional immunostaining after 24 h of treatment. Scale bar: 100 μm. (**D**) Relative protein expression in cell-surface and whole-cell fractions after 24 h of treatment. Dunnett’s test vs. Control, *p < 0.05, **p < 0.01 (n = 3). Original blots are presented in Supplementary Fig. 7.

To elucidate the mechanism by which these compounds increased surface-localized CLDN1 expression, we investigated from two points of view: alteration of protein expression and subcellular localization. By qRT-PCR and immunoblotting, we found that heparinoid significantly increased the mRNA and protein expression of CLDN1, whereas p-Pyr had no such effects (Fig. 3A,B). Conversely, a conventional immunostaining assay including fixation and permeabilization steps suggested that heparinoid increased surface-localized CLDN1 by enhancing total CLDN1 expression in the cells, and p-Pyr promoted the translocation of intracellular CLDN1 to the cell surface (Fig. 3C). Furthermore, similar results were obtained by immunoblotting after the purification of cell surface proteins (Fig. 3D). Heparinoid increased CLDN1 levels in both the cell-surface and whole-cell fractions, resulting in no significant change in the cell-surface/whole-cell abundance ratio, whereas p-Pyr only enhanced CLDN1 levels on the cell surface fraction. Accordingly, it was strongly suggested that heparinoid and p-Pyr enhanced surface-located CLDN1 expression by acting on biosynthesis and translocation pathways, respectively.

### Molecular target of heparinoid

**Figure 4:**
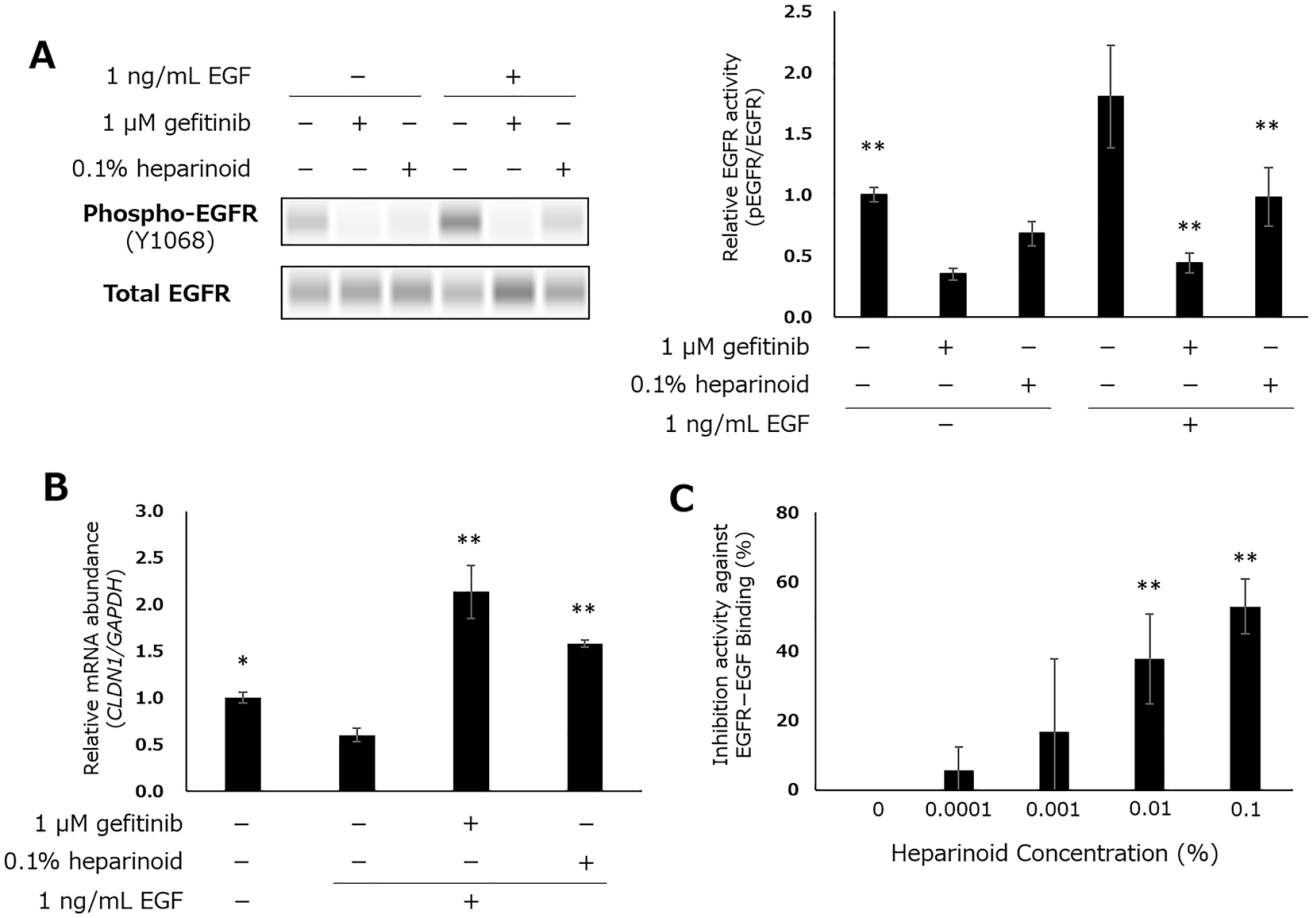
Heparinoid-mediated EGFR inhibition. (**A**) Relative EGFR phosphorylation in EGF-starved NHEKs after 10 min of EGF exposure. Dunnett’s test vs. EGF-treated Control, *p < 0.05, **p < 0.01 (n = 4). Original blots are presented in Supplementary Fig. 8. (**B**) Relative mRNA abundance of *CLDN1* after 6 h of EGF exposure. Dunnett’s test vs. EGF-treated Control, *p < 0.05, **p < 0.01 (n = 4). (**C**) Inhibition of EGFR–EGF binding by heparinoid. Williams’ test vs. 0%, *p < 0.05, **p < 0.01 (n = 3).

Heparinoid, also called mucopolysaccharide polysulfate^16^, is synthesized from chondroitin sulfate by additional polysulfation; thus, it has a high molecular weight and high hydrophilicity. Therefore, it is difficult for heparinoid to enter the cytoplasm passively, and thus, we assumed that heparinoid acted on extracellular factors, such as epidermal growth factor receptor (EGFR). The EGFR signaling pathway is responsible for various functions, such as cellular growth and viability^17^, ^18^, and EGFR activation is also reported to suppress various barrier-related genes, including *CLDN1* in NHEKs^19^. In our experiments, treatment of NHEKs with heparinoid resulted in decreased EGFR phosphorylation (Fig. 4A). Furthermore, heparinoid inhibited EGFR phosphorylation when recombinant human EGF was added exogenously, and its effect was similar to that of gefitinib, a well-known EGFR inhibitor^20^. In addition, EGF-dependent downregulation of *CLDN1* and its restoration by heparinoid and gefitinib were observed (Fig. 4B). In this experiment, to eliminate the possibility that EGF included in the culture medium induced EGFR activation, cells were incubated with medium lacking EGF for 2 h prior to the administration of heparinoid or gefitinib. However, even under this condition, EGFR was slightly phosphorylated, and this phosphorylation was diminished by the addition of heparinoid and gefitinib. Thus, the remaining phosphorylation of EGFR in the starvation medium was assumed to be caused by the autocrine action of the EGFR ligand generated by NHEKs themselves.

From these findings, it was suggested that heparinoid inhibits EGFR phosphorylation and its downstream signaling. Then, to confirm whether heparinoid directly inhibits EGFR–EGF binding, we performed a cell-free in vitro binding assay with EGFR immobilized to the bottom of the microplate and EGF labeled with fluorescein (Fig. 4C). Heparinoid was demonstrated to inhibit EGFR–EGF binding in a concentration-dependent manner. Because 0.1% heparinoid inhibited EGFR–EGF binding by approximately 50%, it is suggested that the heparinoid-dependent enhancement of CLDN1 is caused partially by the inhibition of EGFR–EGF binding,

### Effects of the hit compounds on other TJ components

**Figure 5:**
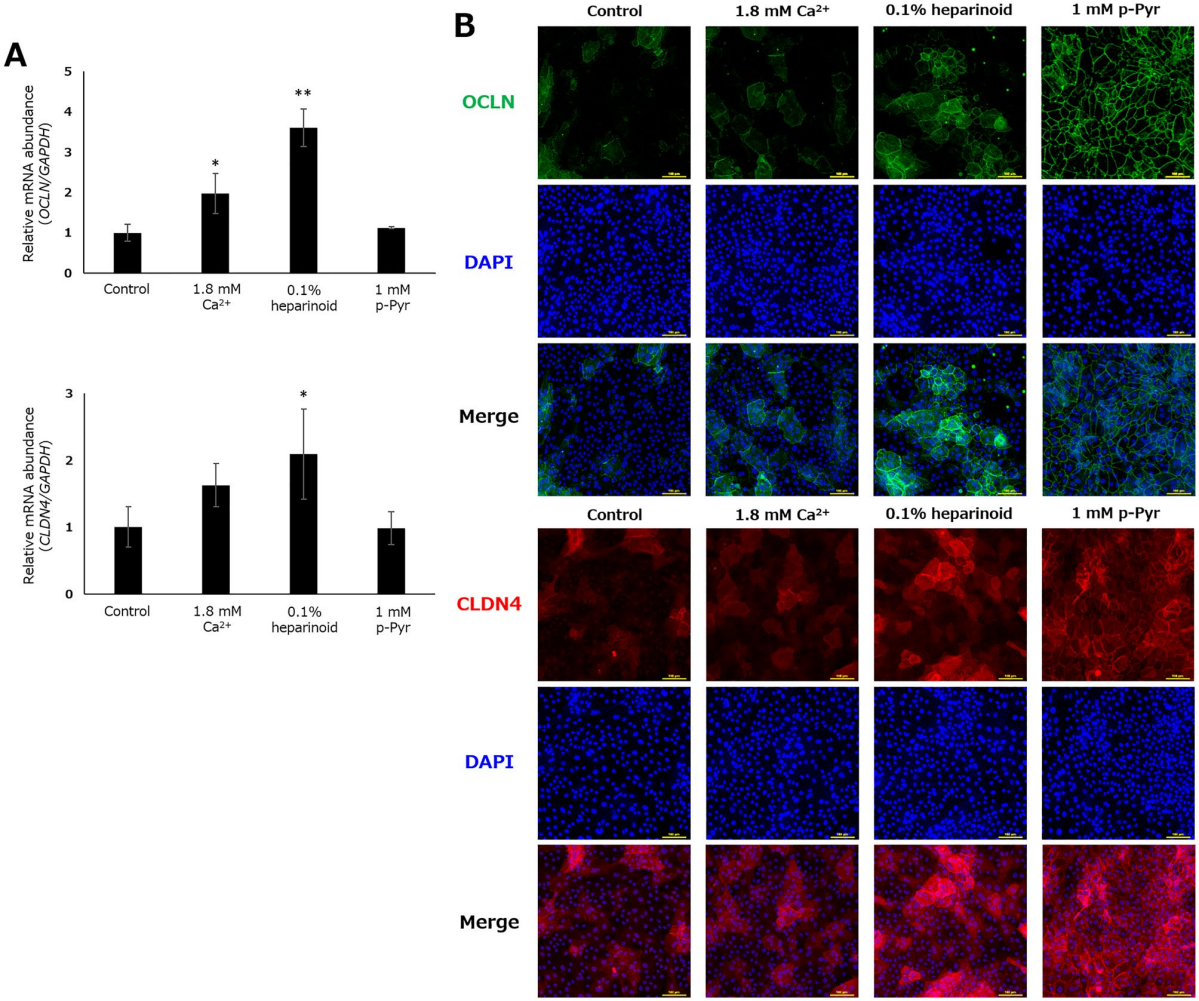
Effects of the hit compounds on OCLN and CLDN4 in NHEKs. (**A**) Relative mRNA abundance after 6 h of treatment. Dunnett’s test vs. Control, *p < 0.05, **p < 0.01 (n = 3). (**B**) Representative confocal microscopic images using conventional immunostaining after 24 h of treatment. Scale bar: 100 μm.

The impact of the hit compounds on TJ components other than CLDN1 was also assessed, confirming that heparinoid also increased *OCLN* and *CLDN4* mRNA transcription (Fig. 5A). Meanwhile, p-Pyr enhanced the plasma membrane localization of *OCLN* and *CLDN4* without altering their mRNA abundance (Fig. 5B). These results strongly support that heparinoid and p-Pyr have the potential to improve the function of TJs by increasing the expression of TJ components and accelerating their translocation to the plasma membrane, respectively.

### Barrier function assay

**Figure 6:**
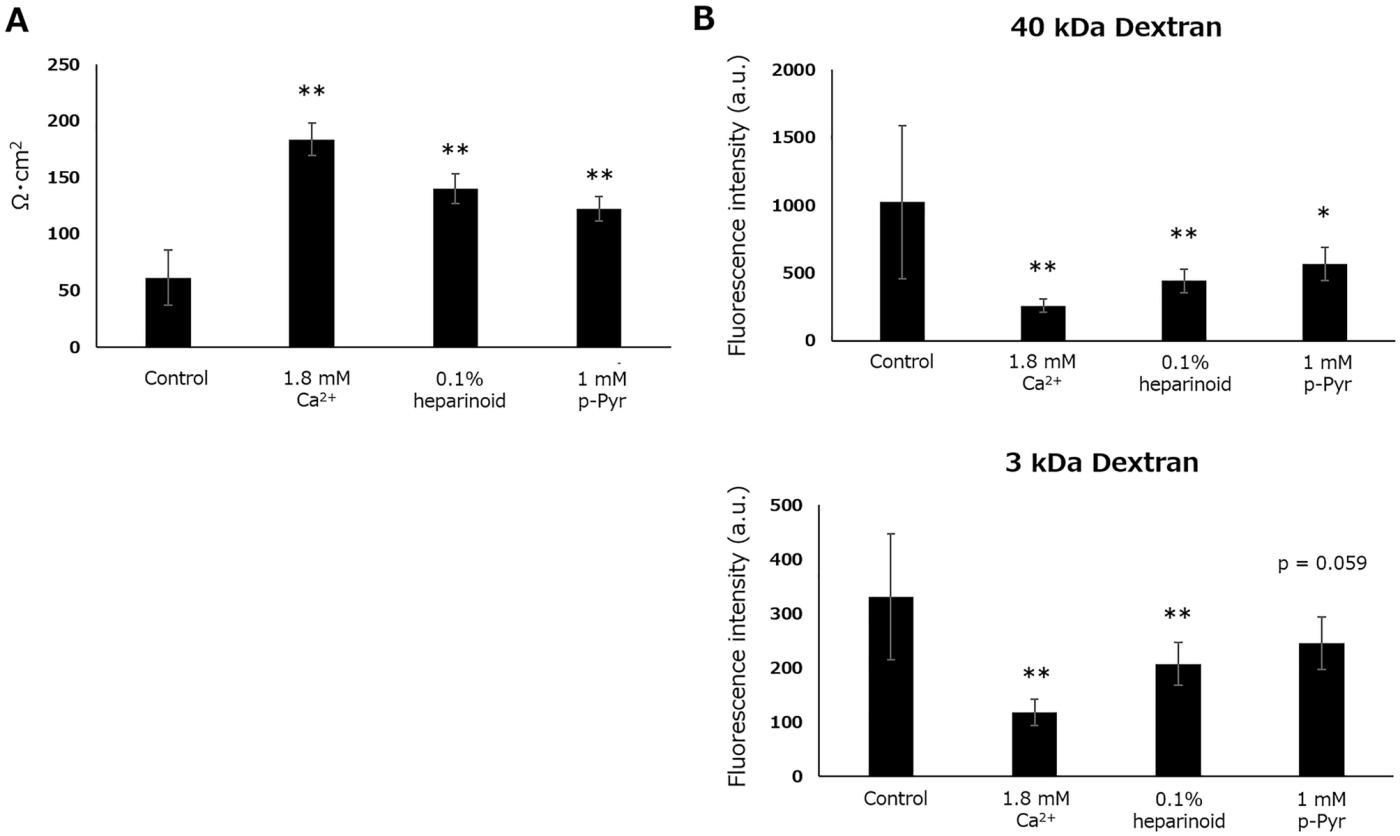
Alteration of barrier function in NHEKs following hit compound treatment. (**A**) TEER after 24 h of treatment. Dunnett’s test vs. Control, *p < 0.05, **p < 0.01 (n = 7–8). (**B**) Permeability of fluorescently labeled dextrans after 24 h of treatment. Dunnett’s test vs. Control, *p < 0.05, **p < 0.01 (n = 7–8).

Because heparinoid and p-Pyr improved TJ function by acting on CLDN1, OCLN, and CLDN4, we also assessed their potential to enhancing barrier function (Fig. 6A). Both heparinoid and p-Pyr significantly increased transepithelial electrical resistance (TEER) in NHEKs. Furthermore, barrier function was assessed by measuring the permeation of fluorescently labeled dextrans (Fig. 6B). Consequently, heparinoid and p-Pyr significantly inhibited the permeation of 40 kDa dextran, whereas only heparinoid significantly suppressed the permeation of 3 kDa dextran.

## Discussion

TJs play an important role as a physical barrier of the skin together with the stratum corneum, and they are essential factors for skin health because of functions such as inhibiting pathogen invasion, retaining water within the skin, promoting the development of a normal stratum corneum by maintaining Ca^2+^ concentrations, and suppressing nerve elongation from deep inside the skin^5,21,22^. In fact, *CLDN1*-deficient mice are reported to die within 1 day of birth, and clinical studies have demonstrated CLDN1 suppression in the case of atopic dermatitis and NISCH syndrome^6–9^. Thus, a number of studies aiming to enhance TJ function have been conducted. However, many of these studies focused on total CLDN1 expression, whereas we focused on compounds that actually reinforce TJ function. In fact, it has been reported that CLDN1 expression decreases with aging, but the same report identified no significant reduction in its mRNA expression^10^. This result suggests that the age-related decrease of CLDN1 originates from increased proteolysis rather than decreased gene expression. Previously, dephosphorylation and LNX1p80-dependent ubiquitylation were described to be involved in the endocytosis and subsequent lysosomal degradation of CLDN1^23,24^. Therefore, it is suggested that the age-related impairment of TJ function is linked to improper control of the subcellular localization of TJ components, and we believe it is crucial to assess TJ function by focusing on the expression of cell surface-localized CLDN1.

To detect cell surface-localized CLDN1 selectively and efficiently, we developed a live-cell immunostaining method that does not include fixation and permeabilization steps. This method was realized by using an antibody capable of recognizing the extracellular domain of CLDN1 without causing plasma membrane damage. However, in this assay system, a slight fluorescence signal was observed from intracellular fraction by microscopic imaging. The anti-CLDN1 antibody clone 7A5 is reported to bind extracellular loop 2 domain of CLDN1 and reduce barrier function in NHEKs, as indicated by decreased TEER and increased dextran permeability^25^. Because CLDN1 internalization may cause decreased barrier function, the fluorescence signal detected the inside the cell might have arisen from CLDN1 originally localized on the cell surface. Cytochalasin D-dependent amelioration of the fluorescence signal from the cell surface supports the aforementioned hypothesis. Additionally, because the ratio of the fluorescence signal between the cell surface and cytoplasm was also increased compared to that in the conventional immunostaining method with a permeabilization step, the signal obtained from inside the cell was not considered a crucial issue for the study purpose.

Using the developed screening system, we identified heparinoid and p-Pyr as hit compounds. Because this method has the potential to easily evaluate larger numbers of samples, it is expected that additional effective compounds will be found in future studies. Heparinoid enhanced the expression of the TJ proteins CLDN1, OCLN, and CLDN4. Because of its high molecular weight and hydrophilicity, heparinoid was considered to act on the extracellular surface. In particular, we assessed the effect on EGFR, a membrane-anchored protein known to regulate *CLDN1* expression. It was confirmed that heparinoid markedly reduced EGFR phosphorylation. Additionally, gefitinib, a widely used EGFR inhibitor, inhibited EGFR phosphorylation and amplified *CLDN1* mRNA expression similarly as heparinoid. Thus, heparinoid’s CLDN1-enhancing effect is believed to be attributable to its inhibitory effects on EGFR. Although EGFR signaling is known to be deeply involved in cell growth and viability, it has also been reported to deteriorate barrier function^19^. In fact, EGFR signaling is known to be involved in the development of skin diseases such as psoriasis^26^. Therefore, although the effect of EGFR signaling on health depends on the situation, heparinoid might be a useful candidate for treating certain skin diseases.

In recent studies, heparinoid was found to increase CLDN1 expression by inhibiting extracellular signal-regulated kinase (ERK)^27,28^. Because ERK is a well-known enzyme activated downstream of EGFR^29^, this finding is consistent with our work. In other words, heparinoid is believed to inhibit EGFR activation, leading to ERK inhibition and the subsequent enhancement of CLDN1 expression. In addition, it was previously demonstrated that chondroitin sulfate, a precursor of heparinoid, inhibits EGFR signaling^30^ and thus we considered it reasonable that heparinoid, which is similar to chondroitin sulfate in chemical structure, inhibited EGFR–EGF binding and the subsequent receptor activation.

Meanwhile, p-Pyr is an active metabolite of pyridoxine, also known as vitamin B6, produced by enzymes such as pyridoxal kinase and pyridoxamine phosphate oxidase^31^. We confirmed that p-Pyr has no potentiating effect on the expression of CLDN1, OCLN, and CLDN4, but it can promote the translocation of these factors to the cell surface. Based on these results, p-Pyr would not have been identified as a hit compound by only evaluating CLDN1 total expression using methods such as ELISA. The translocation of CLDN1 is controlled by various post-translational modifications, such as phosphorylation (S69, S192, and T191 in human) for trafficking to the plasma membrane^11,12^ and nitration or ubiquitylation for internalization^13,24^. Therefore, p-Pyr might act on protein kinase C, protein kinase A, LNX1p80, and nitric oxide synthases involved in these modifications.

**Figure 7:**
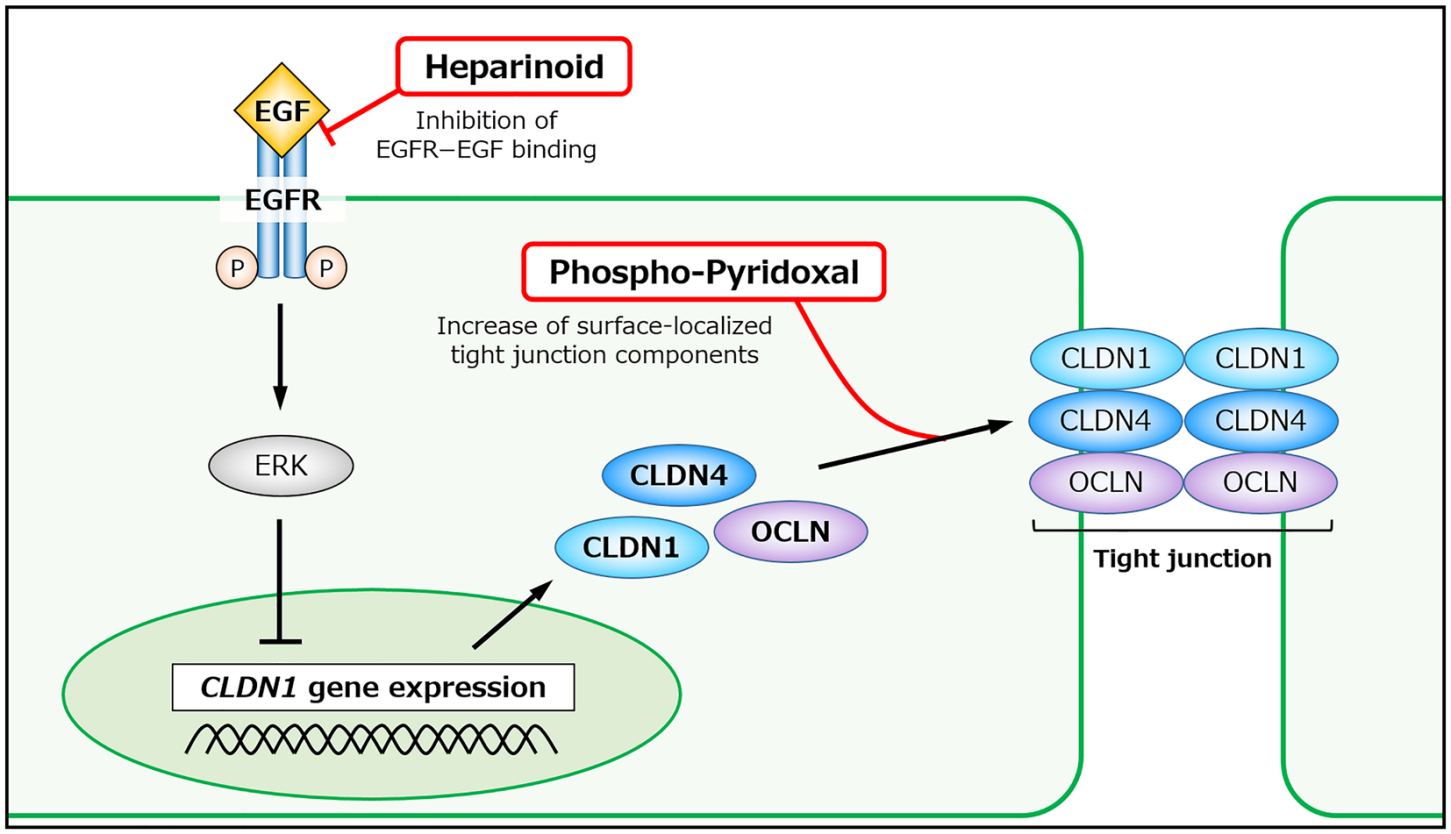
Assumed mechanisms of action of the hit compounds. Schematic diagram of heparinoid- and phospho-pyridoxal-induced improvement in TJ function in keratinocytes.

The two hit compounds identified in the present study were confirmed to increase the abundance of the TJ proteins CLDN1, OCLN, and CLDN4 on the plasma membrane by different mechanisms (Fig. 7). Heparinoid activates the biosynthetic pathway, and p-Pyr promotes the translocation of each component to the cell surface. In fact, it was suggested that these compounds enhanced barrier function in TEER and dextran (40 and 3 kDa) permeation assays in NHEKs. Therefore, heparinoid and p-Pyr are potent candidates for ameliorating various skin symptoms such as dryness and pruritus. Furthermore, because of their different mechanisms, co-administration of these compounds could result in stronger effects on TJ function. However, a limitation of the present study was that all experiments evaluating TJ function were performed using monolayer-cultured keratinocytes. Thus, it is necessary to determine whether hit compounds can elicit the observed functions in clinical research in the future. In addition, the development of novel drug delivery systems could be needed.

## Materials and methods

### Cell culture

NHEKs (KK-4009; Kurabo Industries Ltd., Osaka, Japan) were cultured in HuMedia-KG2 (KK-2150S; Kurabo Industries) containing 10 μg/mL insulin, 0.1 ng/mL human recombinant EGF, 0.67 μg/mL hydrocortisone, 50 μg/mL gentamicin, 50 ng/mL amphotericin B, and 0.4% v/v bovine pituitary extract at 37 °C in a humidified atmosphere of 5% CO_2_. To reduce the impact of EGF in the culture medium in the EGFR inhibition assay, the medium was replaced with EGF-free medium. Two hours after starvation, each test compound was added to the cells.

The reagents added to the cells were as follows: CaCl_2_ (07057-00; Kanto Chemical Co. Inc., Tokyo, Japan), cytochalasin D (034-25881; FUJIFILM WAKO Pure Chemical Corporation, Osaka, Japan), heparinoid (Lot No. DHD171103; Yantai Dongcheng Biochemical Pharmaceutical Group Co., Ltd., Shandong, China), p-Pyr (165-20943; FUJIFILM WAKO Pure Chemical Corporation), Triton^®^-X 100 (04605-250; FUFIFILM WAKO Pure Chemical Corporation), gefitinib (078-06561; FUJIFILM WAKO Pure Chemical Corporation), human recombinant EGF (059-07873; FUJIFILM WAKO Pure Chemical Corporation), HEPES buffer (345-06681; FUJIFILM WAKO Pure Chemical Corporation), sodium pyruvate (190-14881; FUJIFILM WAKO Pure Chemical Corporation), D (+)-glucose (50-99-7; FUJIFILM WAKO Pure Chemical Corporation), NaCl (198-01675; FUJIFILM WAKO Pure Chemical Corporation) and MgCl_2_ (139-00185; FUJIFILM WAKO Pure Chemical Corporation).

### Compound screening with a live-cell immunostaining method

Confluent NHEKs cultured in 96-well plates were incubated with culture medium containing 1.8 mM CaCl_2_ or the test compounds (1 mM or 0.1% w/v) and then cultured for 24 h. The culture media of all the wells contained 0.1% v/v dimethyl sulfoxide. After removing the medium, the cells were washed with PBS. Subsequently, the cells were incubated with anti-CLDN1 antibody clone 7A5 (MABT366; Merck KGaA, Darmstadt, Germany; 1:200 dilution in PBS containing 0.1% w/v bovine serum albumin) for 2 h at 25 °C followed by three washes with PBS. Then, the cells were incubated with Alexa Fluor^®^ 488-conjugated anti-mouse IgG (ab150113; Abcam, Cambridge, UK; 1:1000 dilution) for 2 h at 25 °C. After three washes with PBS, 100 μL of PBS were added, and the fluorescence intensity was measured using a microplate reader (BioTek Synergy H1, Agilent Technologies, Santa Clara, CA, USA; Ex 485 nm/Em 526 nm). To reduce variation in the fluorescence intensity attributable to the position within the well, five points (center, upper, lower, left, and right) were read and averaged in each well.

To exclude the possibility of false-positive results, microscopic imaging of the cells was performed. The medium was replaced with PBS containing DAPI (227549; Abcam; 1:3000 dilution), and the cells were observed by confocal laser microscopy (A1R HD25; Nikon Corporation, Tokyo, Japan; Ex 345 nm/Em 455 nm and Ex 405 nm/Em 519 nm).

### Cytotoxicity assay

Confluent NHEKs cultured in 96-well plates were incubated with media containing 1.8 mM CaCl_2_, 0.1% w/v heparinoid, 1 mM p-Pyr or 1% v/v Triton^®^ X-100 and cultured for 24 h. The LDH assay was performed using the culture media and LDH Cytotoxicity Assay Kit (18250-64; NACALAI TESQUE, INC., Kyoto, Japan) following the manufacturer’s protocols. The signals obtained from vehicle- and Triton^®^ X-100- treated samples were defined as 0% and 100% cytotoxicity, respectively.

### Imaging of TJ components by conventional immunostaining

Confluent NHEKs cultured in 96-well plates were washed with PBS and then exposed to methanol (− 20 °C) for 10 min. The cells were washed with PBS and blocked with PBS containing 0.1% w/v bovine serum albumin for 1 h. Then, the cells were incubated with anti-CLDN1 (#13255; Cell Signaling Technology, Inc., Danvers, MA, USA), anti-OCLN (OC-3F10; Thermo Fisher Scientific, Inc., Waltham, MA, USA), and anti-CLDN4 (ab210796; Abcam), all with a 1:1000 dilution, for 16 h at 4 °C. After three washes with PBS, cells were incubated with secondary antibody (Alexa Fluor^®^ 488-conjugated anti-mouse IgG [150113; Abcam] or Alexa Fluor^®^ 594-conjugated anti-rabbit IgG [150080; Abcam]; 1:1000 dilution) for 2 h. After three washes with PBS, DAPI-containing PBS was added to the cells, and fluorescent imaging was performed by confocal laser microscopy (Ex 345 nm/Ex 455 nm, Ex 405 nm/Em 510 nm, and Ex 590 nm/Em 615 nm).

### Gene expression analysis

RNA extraction from the cells and subsequent cDNA synthesis were performed using a Superprep^®^ Cell Lysis and RT Kit for qPCR (SCQ-101; TOYOBO Co., Ltd., Osaka, Japan) following the manufacturer’s protocols. Then, gene expression was evaluated using KOD SYBR^®^ qPCR Mix (QKD-201; TOYOBO Co., Ltd.) and a thermal cycler (CFX96 Real-Time System; Bio-Rad Laboratories, Inc., Hercules, CA, USA). The primer sequences were as follows: human *CLDN-1* forward, 5′-gtctttgactccttgctgaatctg-3′; human *CLDN-1* reverse, 5′-cacctcatcgtcttccaagcac-3′; human *OCLN* forward, 5′-atggcaaagtgaatgacaagcgg-3′; human *OCLN* reverse, 5′-ctgtaacgaggctgcctgaagt-3′; human *CLDN-4* forward, 5′-agtgcaaggtgtacgactcgct-3′; human *CLDN-4* reverse, 5′-cgctttcatcctccaggcagtt-3′; human *GAPDH* forward, 5′-gtctcctctgacttcaacagcg-3′; and human *GAPDH* reverse, 5′-accaccctgttgctgtagccaa-3′.

### Immunoblotting

NHEKs cultured in 48-well plates were washed with ice-cold PBS and then lysed via incubation with RIPA buffer containing protease inhibitor cocktail (08714-04; NACALAI TESQUE, INC.) on ice for 10 min. For the EGFR inhibition assay, Phosstop™ (4906845001; Merck KGaA) was added to the lysis buffer. After centrifugation at 15,000×*g*, supernatant was collected and applied to an automated western blot analyzer (Abby; Bio-Techne Corporation, Minneapolis, MN, USA). Anti-CLDN1 antibody (ab15098; Abcam), anti-pEGFR (Y1068) antibody (#2234; Cell Signaling Technology), and anti-EGFR antibody (#4267; Cell Signaling Technology) were used.

### Extraction of cell surface protein

NHEKs cultured in 12-well plates were washed twice with ice-cold PBS and then biotinylated using a Pierce™ Cell Surface Protein Isolation Kit (89881; Thermo Fisher Scientific) following the manufacturer’s protocols. Lysis buffer without sodium dodecyl sulfate (SDS) was prepared using RIPA buffer containing protease inhibitor cocktail to prevent interference with subsequent biotin–streptavidin binding. Cells were lysed by adding the aforementioned buffer and rocking on ice for 1 h. After centrifugation at 15,000×*g*, the supernatant was collected and mixed with NeutrAvidin Agarose (contained in the Cell Surface Protein Isolation Kit) for 1 h at 25 °C. NeutrAvidin Agarose was washed four times with Wash Buffer (contained in the kit) and heated for 5 min at 95 °C in the presence of 2% w/v SDS and 50 mM dithiothreitol to elute cell surface-localized proteins from the agarose. NeutrAvidin Agarose-purified and non-purified samples were analyzed as the cell-surface and whole-cell fractions, respectively. To evaluate the ratio of plasma membrane localization of CLDN1, the cell-surface fraction/whole-cell fraction expression ratio was calculated.

### EGFR–EGF binding assay

Fluorescein-labeled EGF (Fluo-EGF) was prepared by mixing 100 μM human recombinant EGF (053-07871; FUJIFILM WAKO Pure Chemical Corporation) and 1 mM fluorescein 5-isothiocyanate (FITC; F0026; Tokyo Chemical Industry Co., Ltd., Tokyo, Japan) in 100 mM NaHCO_3_ aq for 16 h at 25 °C, followed by the removal of unreacted FITC with PD SpinTrap™ G-25 (28918004; Cytiva, Tokyo, Japan). The proportion of fluorescein-labeled EGF molecules among all molecules was calculated to 0.16.

To immobilize EGFR to the bottom of the microplate, 100 μL of 10 μg/mL human Fc region-fused human recombinant EGFR (10001-H02H; Sino Biological, Inc., Beijing, China) in PBS were added to Pierce™ Protein A Coated Plate (15132; Thermo Fisher Scientific), followed by 1 h of reaction at 25 °C. After three washes with ice-cold PBS, 100 μL of 100 ng/mL Fluo-EGF in PBS were added, followed by reaction for 1 h at 25 °C. After washing with PBS, The fluorescence intensity was measured (Ex 485 nm/Em 526 nm). The signals obtained in the absence and presence of EGFR were defined as 0% and 100% EGFR–EGF binding, respectively.

### TEER assay

Permeable Polyester Membrane Inserts (07-200-154; Corning Inc., Corning, NY, USA) were loaded on a 24-well plates, and NHEKs were cultured on the insert until reaching confluency. After reaching confluency, cells were additionally cultured for 4 days and then incubated with 1.8 mM Ca^2+^-containing medium for 24 h to initiate differentiation. Then, the media were removed, and Ca^2+^- or hit compounds-containing media were added. After 24 h of incubation, TEER was measured using a Millicell^®^-ERS Voltohmmeter (MERS00002; Merck KGaA), and the value multiplied by 0.3 cm^2^ of the cell-cultured area was calculated as the electrical resistance per unit area (Ω·cm^2^).

### Dextran permeation assay

After the TEER assay, dextran solution (10 mM HEPES [pH 7.4], 1 mM sodium pyruvate, 1 mM CaCl_2_, 10 mM glucose, 145 mM NaCl, 1 mM MgCl_2_, 0.33 mg/mL fluorescein-labeled dextran, 40 kDa [D1844; Thermo Fisher Scientific], 0.33 mg/mL Texas Red-labeled dextran, 3 kDa [D3329; Thermo Fisher Scientific]) was added to upper chamber. After 2 h of incubation at 37 °C, the solution of lower chamber was collected, and fluorescence intensity was measured using microplate reader (Ex 488 nm/Em 528 nm and Ex 590 nm/Em 615 nm).

### Statistics

Statistical analysis software JMP^®^ (JMP Statistical Discovery LLC, Cary, NC, USA) was used to perform Student’s *t*-test and Dunnett’s test to determine significances between the treated and control groups. Another software Pharmaco Basic ver. 16 (Scientist Press Co. Ltd., Tokyo, Japan) was used to perform William’s test to evaluate the dose-dependency of heparinoid’s efficacy. Data are presented as mean ± standard deviation and “n” depicted the number of biological replicates in each experiment.

### Supplementary Information

Supplementary Information.

## Data Availability

The dataset used and/or analyzed during the current study are available from the corresponding author (S.Y.) on reasonable request.

## References

[1] Proksch E, Brandner JM, Jensen JM (2008). The skin: An indispensable barrier. Exp. Dermatol..

[2] Kabashima K, Honda T, Ginhoux F, Egawa G (2019). The immunological anatomy of the skin. Nat. Rev. Immunol..

[3] Madison KC (2003). Barrier function of the skin: “La raison d’être” of the epidermis. J. Investig. Dermatol..

[4] Bäsler K, Brandner JM (2017). Tight junctions in skin inflammation. Pflugers Arch..

[5] Takahashi S (2019). Homeostatic pruning and activity of epidermal nerves are dysregulated in barrier-impaired skin during chronic itch development. Sci. Rep..

[6] Furuse M (2002). Claudin-based tight junctions are crucial for the mammalian epidermal barrier: A lesson from claudin-1-deficient mice. J. Cell Biol..

[7] De Benedetto A (2011). Tight junction defects in patients with atopic dermatitis. J. Allergy Clin. Immunol..

[8] Yuki T, Tobiishi M, Kusaka-Kikushima A, Ota Y, Tokura Y (2016). Impaired tight junctions in atopic dermatitis skin and in a skin-equivalent model treated with interleukin-17. PLoS One.

[9] Hadj-Rabia S (2004). Claudin-1 gene mutations in neonatal sclerosing cholangitis associated with ichthyosis: A tight junction disease. Gastroenterology.

[10] Jin SP (2016). Changes in tight junction protein expression in intrinsic aging and photoaging in human skin in vivo. J. Dermatol. Sci..

[11] French AD (2009). PKC and PKA phosphorylation affect the subcellular localization of claudin-1 in melanoma cells. Int. J. Med. Sci..

[12] Marunaka K, Kobayashi M, Shu S, Matsunaga T, Ikari A (2019). Brazilian green propolis rescues oxidative stress-induced mislocalization of Claudin-1 in human keratinocyte-derived HaCaT cells. Int. J. Mol. Sci..

[13] Kobayashi M, Shu S, Marunaka K, Matsunaga T, Ikari A (2020). Weak ultraviolet B enhances the mislocalization of Claudin-1 mediated by nitric oxide and peroxynitrite production in human keratinocyte-derived HaCaT cells. Int. J. Mol. Sci..

[14] Fukasawa M (2015). Monoclonal antibodies against extracellular domains of claudin-1 block hepatitis C virus infection in a mouse model. J. Virol..

[15] Yuki T (2007). Tight junction proteins in keratinocytes: Localization and contribution to barrier function. Exp. Dermatol..

[16] Fujiwara-Sumiyoshi S (2021). Mucopolysaccharide polysulfate promotes microvascular stabilization and barrier integrity of dermal microvascular endothelial cells via activation of the angiopoietin-1/Tie2 pathway. J. Dermatol. Sci..

[17] Gibbs S (2000). Epidermal growth factor and keratinocyte growth factor differentially regulate epidermal migration, growth, and differentiation. Wound Repair Regen..

[18] Rodeck U (1997). EGF-R dependent regulation of keratinocyte survival. J. Cell Sci..

[19] Tran QT (2012). EGFR regulation of epidermal barrier function. Physiol. Genom..

[20] Herbst RS, Fukuoka M, Baselga J (2004). Gefitinib—A novel targeted approach to treating cancer. Nat. Rev. Cancer.

[21] Kurasawa M, Maeda T, Oba A, Yamamoto T, Sasaki H (2011). Tight junction regulates epidermal calcium ion gradient and differentiation. Biochem. Biophys. Res. Commun..

[22] Sugawara T (2013). Tight junction dysfunction in the stratum granulosum leads to aberrant stratum corneum barrier function in claudin-1-deficient mice. J. Dermatol. Sci..

[23] Fujii N (2016). Hypotonic stress-induced down-regulation of Claudin-1 and -2 mediated by dephosphorylation and clathrin-dependent endocytosis in renal tubular epithelial cells. J. Biol. Chem..

[24] Takahashi S (2009). The E3 ubiquitin ligase LNX1p80 promotes the removal of claudins from tight junctions in MDCK cells. J. Cell Sci..

[25] Nakajima M (2015). Claudin-1 binder enhances epidermal permeability in a human keratinocyte model. J. Pharmacol. Exp. Ther..

[26] Wang S, Zhang Z, Peng H, Zeng K (2019). Recent advances on the roles of epidermal growth factor receptor in psoriasis. Am. J. Transl. Res..

[27] Koda A (2023). The effects of mucopolysaccharide polysulfate on steroid-induced tight junction barrier dysfunction in human epidermal keratinocytes and a 3D skin model. Skin Pharmacol. Physiol..

[28] Fujikawa M (2022). Effects of mucopolysaccharide polysulphate on tight junction barrier in human epidermal keratinocytes. Exp. Dermatol..

[29] Avraham R, Yarden Y (2011). Feedback regulation of EGFR signalling: Decision making by early and delayed loops. Nat. Rev. Mol. Cell Biol..

[30] Kitazawa K, Nadanaka S, Kadomatsu K, Kitagawa H (2021). Chondroitin 6-sulfate represses keratinocyte proliferation in mouse skin, which is associated with psoriasis. Commun. Biol..

[31] Van den Eynde MDG, Scheijen JLJM, Stehouwer CDA, Miyata T, Schalkwijk CG (2021). Quantification of the B6 vitamers in human plasma and urine in a study with pyridoxamine as an oral supplement; pyridoxamine as an alternative for pyridoxine. Clin. Nutr..

