# Supplementary material for: Development of tight junction-strengthening compounds using a high-throughput screening system to evaluate cell surface-localized claudin-1 in keratinocytes
Source: Sci Rep. 2024 Feb 9;14:3312. doi: 10.1038/s41598-024-53649-1 (PMC10853544; doi:10.1038/s41598-024-53649-1)
Supplement: Supplementary file 1 — Supplementary Information. [file 41598_2024_53649_MOESM1_ESM.pdf]

Development of tight junction-strengthening compounds using a high-throughput screening system to evaluate cell surface-localized claudin-1 in keratinocytes

Hiroki Sakamoto, Momoyo Nishikawa, Seigo Yamada*

Research & Development Headquarters, Well-being Research Laboratories, Lion Corporation, 100 Tajima, Odawara-shi, Kanagawa, 256-0811, Japan

*email: seigo-y@lion.co.jp

**Tested compounds** (added to NHEKs at concentrations of 1 mM or 0.1% w/v)			
Ufenamate	Panthenol	Sulfamine	Chlorpheniramine
Dexamethasone	Calcium D-pantothenate	Sulfamethoxazole	Diphenhydramine
Dexamethasone acetate	Retinol	Sulfixoxazole	Cetylpyridinium
Hydrocortisone	Retinol acetate	Homosulamine	Benzalkonium chloride
Hydrocortisone acetate	Vitamin A oil	Crotamiton	Benzyl alcohol
Hydrocortisone butyrate	Pyridoxine	Nonivamide	Benzethonium chloride
Predonisolone	Phospho pyridoxal	Camphor	Nicotine
Predonisolone acetate	Pyridoxal	Capsaicin	Resolcinol
Betamethasone Valerate	Pyridoxamine	Dibucaine	Acrinol
Glycyrrhetinic acid	Nicotinic acid	Procaine	Benzoic acid
Dipotassium glycyrrhizinate	Nicotinamide	Lidocaine	Citric acid
Allantoin	Benzyl Nicotinate	Ethyl aminobenzoate	Alminium silicate
Lactic acid	Fradiomycin	Heparin sodium	Talc
Ascorbic acid	Oxytetracycline	Heparinoid	Zinc chloride
Ergocalciferol	Tetracycline	Hyaluronic acid	
Tocopherol	Chloramphenicol	Glycerin	
Tocopherol succinate	Sulfadiazine	Urea	

Supplementary table

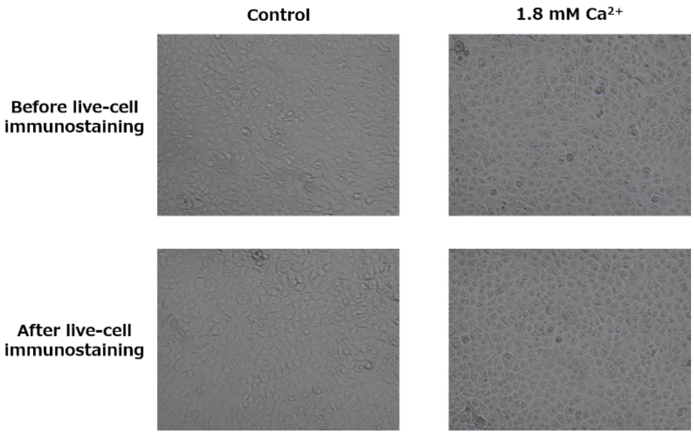

Supplementary figure 1.

Microscopic images of NHEKs before and after live-cell immunostaining.

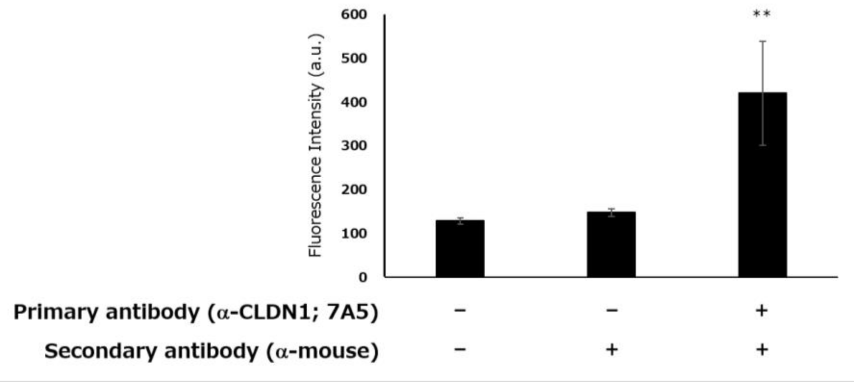

Supplementary figure 2.

Fluorescence intensity of NHEKs in the presence and absence of each antibody. Dunnett's test vs. Primary antibody (−)/Secondary antibody (+), *$p < 0.05$, **$p < 0.01$ ($n = 3$-4).

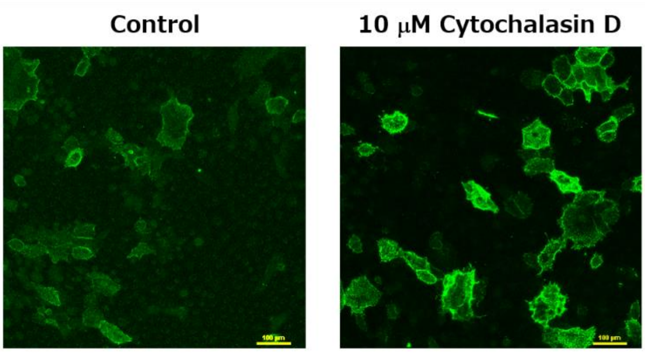

Supplementary figure 3.

Microscopic images of NHEKs in the presence or absence of cytochalasin D. Cells were treated with cytochalasin D 15 min before adding primary antibody.

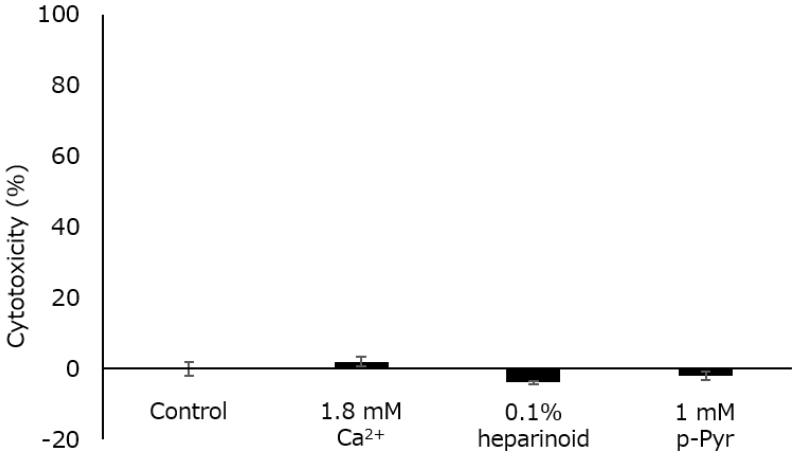

Supplementary figure 4.

Cytotoxicity assay after 24 h hit compounds exposure in NHEKs.

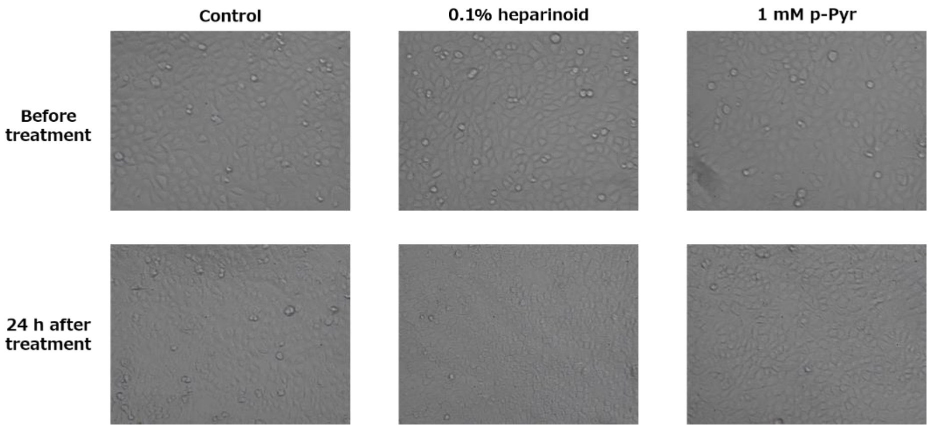

Supplementary figure 5.

Microscopic images of NHEKs before and after treatment of hit compounds

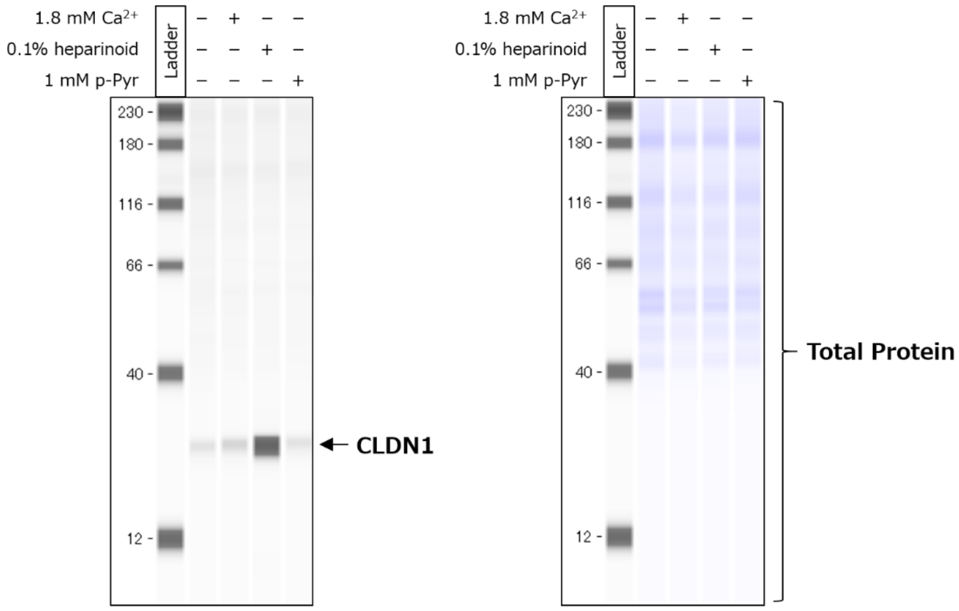

Supplementary figure 6.

Original immunoblotting data of Figure 3B

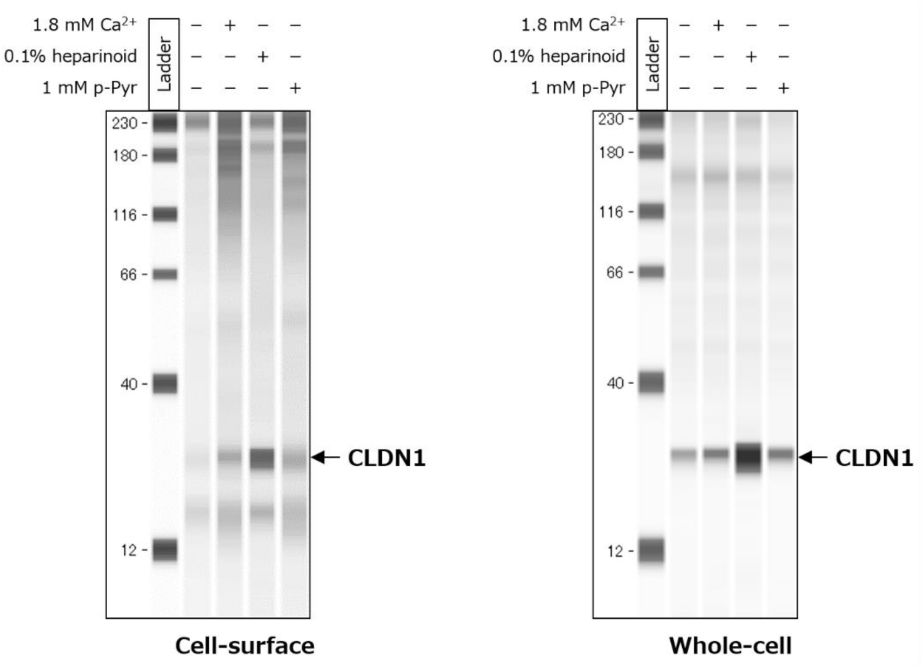

Supplementary figure 7.

Original immunoblotting data of Figure 3D

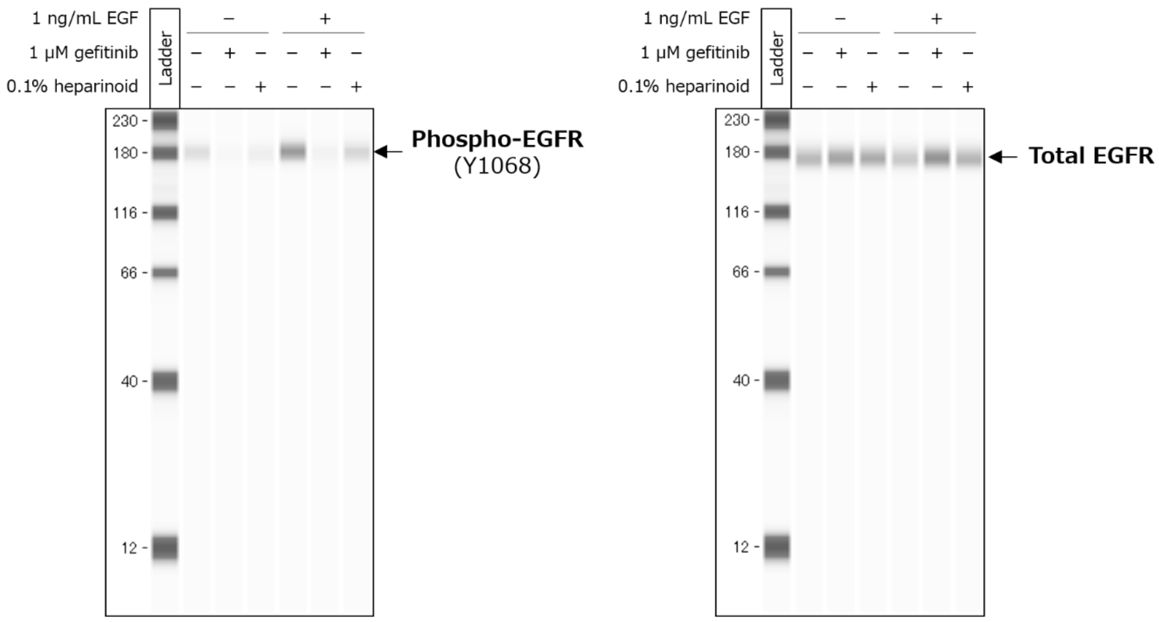

Supplementary figure 8.

Original immunoblotting data of Figure 4A
